# Characterization of Cardiopulmonary Interactions and Exploring Their Prognostic Value in Acute Bronchiolitis: A Prospective Cardiopulmonary Ultrasound Study

**DOI:** 10.3390/tomography8010012

**Published:** 2022-01-05

**Authors:** Moises Rodriguez-Gonzalez, Patricia Rodriguez-Campoy, Ana Estalella-Mendoza, Ana Castellano-Martinez, Jose Carlos Flores-Gonzalez

**Affiliations:** 1 Pediatric Cardiology Division, Puerta del Mar University Hospital, 11010 Cadiz, Spain; doctormoisesrodriguez@gmail.com; 2 Pediatric Intensive Care Unit, Puerta del Mar University Hospital, 11010 Cadiz, Spain; p.r.campoy@gmail.com (P.R.-C.); anaestalella@gmail.com (A.E.-M.); carlosflogon@gmail.com (J.C.F.-G.); 3 Pediatric Nephrology Division, Puerta del Mar University Hospital, 11010 Cadiz, Spain

**Keywords:** acute bronchiolitis, lung ultrasound, echocardiography, point of care ultrasonography, cardiopulmonary ultrasound, cardiopulmonary interactions, pulmonary hypertension, myocardial strain, NT-proBNP

## Abstract

We aimed to delineate cardiopulmonary interactions in acute bronchiolitis and to evaluate the capacity of a combined cardiopulmonary ultrasonography to predict the need for respiratory support. This was a prospective observational single-center study that includes infants <12 month of age admitted to a hospital due to acute bronchiolitis. All the included patients underwent clinical, laboratory and cardiopulmonary ultrasonographic evaluation at the same time point within 24 h of hospital admission. The existence of significant correlation between cardiac and respiratory parameters was the primary outcome. The association of different cardiopulmonary variables with the need of respiratory support higher than O_2_, the length of stay hospitalization, the PICU stay and the duration of respiratory support were a secondary outcome. We enrolled 112 infants (median age 1 (0.5–3) months; 62% males) hospitalized with acute bronchiolitis. Increased values of the pulmonary variables (BROSJOD score, pCO_2_ and LUS) showed moderate correlations with NT-proBNP and all echocardiographic parameters indicative of pulmonary hypertension and myocardial dysfunction (Tei index). Up to 36 (32%) infants required respiratory support during the hospitalization. This group presented with higher lung ultrasound score (*p* < 0.001) and increased values of NT-proBNP (*p* < 0.001), the Tei index (*p* < 0.001) and pulmonary artery pressures (*p* < 0.001). All the analyzed respiratory and cardiac variables showed moderate-to-strong correlations with the LOS of hospitalization and the time of respiratory support. Lung ultrasound and echocardiography showed a moderate-to-strong predictive accuracy for the need of respiratory support in the ROC analysis, with the AUC varying from 0.74 to 0.87. Those cases of bronchiolitis with a worse pulmonary status presented with a more impaired cardiac status. Cardiopulmonary ultrasonography could be a useful tool to easily identify high-risk populations for complicated acute bronchiolitis hospitalization.

## 1. Introduction

Bronchiolitis is the viral lower respiratory infection that most often affects children under 2 years of age, with a peak of incidence and morbidity in young infants (under 3 months of age) [1,2]. It is characterized by small airway inflammation, edema, mucus production and necrosis, leading to the obstruction of distal bronchioles and pulmonary air trapping with subsequent acute impairment in the gas exchange between the lungs and blood, resulting in hypoxia with or without hypercapnia and secondary acidosis. Of note, approximately 5%–10% of infants with acute bronchiolitis develop acute respiratory failure (ARF), requiring respiratory support and PICU admission [1,2].

The existence of a physiological interdependence between the cardiovascular and respiratory systems has long been recognized. The heart and lungs are anatomically coupled through the pulmonary circulation and coexist within the sealed thoracic cavity, making the function of these systems highly interdependent. During ARF, these interactions are magnified, with a striking effect on the pulmonary vasculature and myocardial function [3,4]. An understanding of the interplay between the cardiac and respiratory systems could be important in the management of severe bronchiolitis. However, the presence and the clinical consequences of adverse cardiopulmonary interactions (CPI) in common respiratory diseases, such as bronchiolitis, remain incompletely understood and poorly studied.

Clinical scores have proven to be inaccurate in predicting a prognosis in acute bronchiolitis, with weak interobserver reliability and limited construct validity [5]. Thus, lung ultrasound (LUS) is now emerging as a valuable tool in the assessment of the respiratory status in bronchiolitis. The literature supports the use of LUS as a reliable imaging test that could benefit the clinical management of bronchiolitis [6,7,8]. The presence of myocardial strain assessed by echocardiography is also being increasingly recognized in this setting, where approximately 20% of patients present myocardial dysfunction (MD) or pulmonary hypertension (PHT) at the time of hospitalization [9]. The integrative cardiopulmonary ultrasound (CPU) approach is an emerging noninvasive technique that combines lung ultrasound (LU) and echocardiography to evaluate lung and cardiac conditions simultaneously, especially in a critical care setting [10,11]. To date, there have been few investigations evaluating the concomitant dysfunction of the pulmonary and cardiac systems in critically ill children [12] and no publications in infants with acute bronchiolitis. Using bedside CPU would allow for a rapid and thorough assessment of both the cardiovascular and respiratory states in these settings. We hypothesized that patients with a more severe respiratory status will present with higher rates of pulmonary hypertension and myocardial dysfunction. Although these findings could be theoretically plausible, there is still scarce evidence documenting CPI and their prognostic implications in this setting, and there are no publications using CPU as a noninvasive tool for a prognosis assessment in bronchiolitis.

The primary objective of this study was to explore the influence of the respiratory state over the heart and pulmonary circulation using CPU in infants with bronchiolitis at early stages of the disease. The secondary objective was to evaluate the prognostic implications of adverse CPI and the predictive values of different CPU measurements in this setting.

## 2. Materials and Methods

### 2.1. Design, Setting and Study Population

We conducted a single center, prospective and observational study from October 2018 to March 2020 at the Pediatric Department of a tertiary university hospital in Cadiz, Spain. We included infants hospitalized with acute bronchiolitis. The diagnosis and management were made by the attending pediatrician following the current international recommendations [13]. The members of the study team were fully independent from establishing a diagnosis. Some of the investigators work at PICU and took care of some of the patients included but always after the diagnosis of acute bronchiolitis by the attending pediatrician and the inclusion of the patient in the study. Exclusion criteria were chronic lung diseases, congenital heart diseases, immunodeficiency, previous cardiopulmonary resuscitation, detection of bacterial superinfection, initiation of respiratory support or the administration of epinephrine or intravenous fluid before the research intervention, incomplete research intervention or medical records and poor-quality ultrasound images. The local ethics committee approved the protocol, and informed parental consent was obtained from all patients.

### 2.2. Research Intervention

After the enrolment of patients, the attending pediatrician and nursery and the sonographers coordinated to perform a clinical evaluation, blood extraction for laboratory analysis and CPU at the same time point within the first 24 h of hospitalization.

#### 2.2.1. Pulmonary Status Assessment

**Figure 1:**
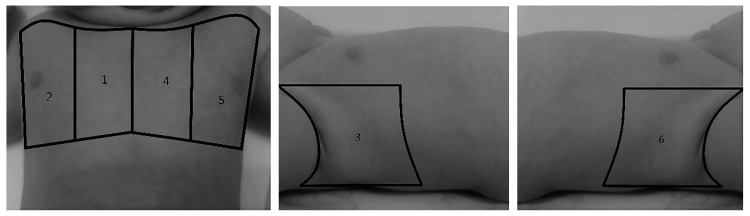
Chest projections used in our study protocol. (1) Right anteromedial (from the parasternal line to right mid-clavicular line), (2) right anterolateral (from the right mid-clavicular line to right anterior axillary line), (3) right lateral (from the right anterior axillary line to right posterior axillary line), (4) left anteromedial (from the parasternal line to left mid-clavicular line), (5) left anterolateral (from the left mid-clavicular line to left anterior axillary line) and (6) left lateral (from the left anterior axillary line to left posterior axillary line).

**Figure 2:**
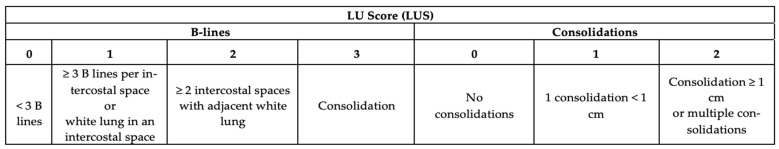
Lung ultrasound score (LUS): based on the number of B-lines, extension of the B-lines and the presence of subpleural consolidation. A score from 0 to 5 was given for each projection in each hemithorax for a maximum LUS of 30 points.

**Figure 3:**
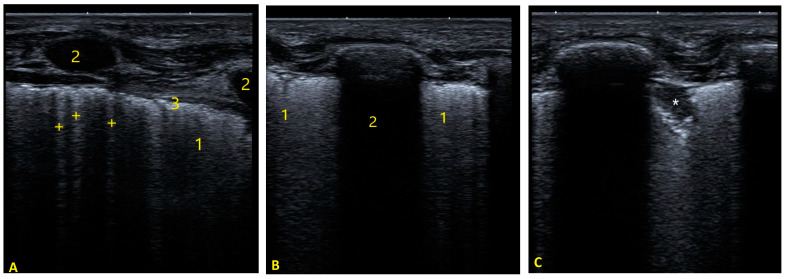
Linear transducer lung ultrasound images showing: (**A**) Left-side panel: B-lines. In this image, multiple B-lines (+) are observed in the intercostal space, some coalescing, giving rise to a white lung image. Irregular and poorly defined pleuropulmonary line. (1) Coalescing b-lines, white lung, (2) ribs and (3) pleuropulmonary line. (**B**) Central panel: Coalescing b-lines or white lung (1) in two adjacent intercostal spaces delimited by a rib (2). (**C**) Right-side panel: Subpleural consolidation. Hypoechoic image with frayed edges (*).

The respiratory state was clinically evaluated by the attending pediatrician using the bronchiolitis of Sant Joan de Déu (BROSJOD) score [14]. This score includes five parameters: heart rate, respiratory rate, SpO_2_, pulmonary auscultation and costal retractions. It ranges from 0 to 14 points. A score greater than 10 points is defined as severe bronchiolitis. The venous pH and pCO_2_ levels were used as laboratory markers of the respiratory state. A significant hypercarbia was defined as pCO_2_ > 55 cmH_2_O independently of the pH levels. Lung ultrasound (LU) was performed by 3 intensive care pediatricians with at least 2 years of experience in LU in routine practice, according to the recommendations for point of care lung ultrasound by Volpicelli et al. [15]. Sonographic examinations were obtained in the supine position in calmed patients without the need for sedation during the procedure. A Doppler ultrasound machine (SonoScape S8, SonoScape Medical Corp., Madrid, Spain) equipped with a high-frequency (16–4 MHz) linear transducer was used to explore 3 chest projections in each hemithorax (Figure 1). We did not evaluate posterior/paravertebral projections due to the difficulty in performing them in these patients, which makes them not very feasible in clinical practice, and furthermore, we believe that these would only provide limited information, since the posterior fields are the ones most frequently affected during the initial phases of acute bronchiolitis, probably due to the supine position of these patients. However, the involvement of the anterior lung fields may reflect greater severity and worse evolution, as shown by the study by Bueno-Campaña et al. [16]. Each area was longitudinally explored in a cephalocaudal direction, evaluating each intercostal space. In each area, the presence of B-lines and subpleural consolidations were analyzed using a lung ultrasound score (LUS). LU studies were recorded and reviewed offline by 1 observer, who was blinded to the patient’s clinical profile. Each area was scored based on the highest scores obtained from the exploration of each intercostal space of the same area. For example, if, in area 1, in an intercostal space, 2 points were obtained, and in another intercostal space of the same area, 3 points were obtained, area 1 was scored with 3 points (Figure 2 and Figure 3). A higher LUS means a more severe pulmonary involvement. A LUS > 10 was considered a moderate-to-severe pulmonary disease in this study. Pleural line abnormalities were not analyzed, as they add little value to consolidations and interstitial syndrome with respect to the assessment of acute bronchiolitis severity and prognosis [16,17].

#### 2.2.2. Cardiac Status Assessment

NT-proBNP was used as a laboratory parameter of myocardial strain. The serum NT-proBNP levels were determined within 2 h of the venous blood samples collection using the electrochemical luminescence immunoassay Elecsys proBNP II (Roche Diagnostics, Sant Cugas del Valles, Barcelona, Spain; 5 pg/mL of the limit of detection, 1.5% intra-run variation; less than 3% inter-run variation). We used the previously reported 99th percentile for infants aged less than 1 year old (1121 pg/mL) as the limit to establish that NT-proBNP was elevated [18].

**Figure 4:**
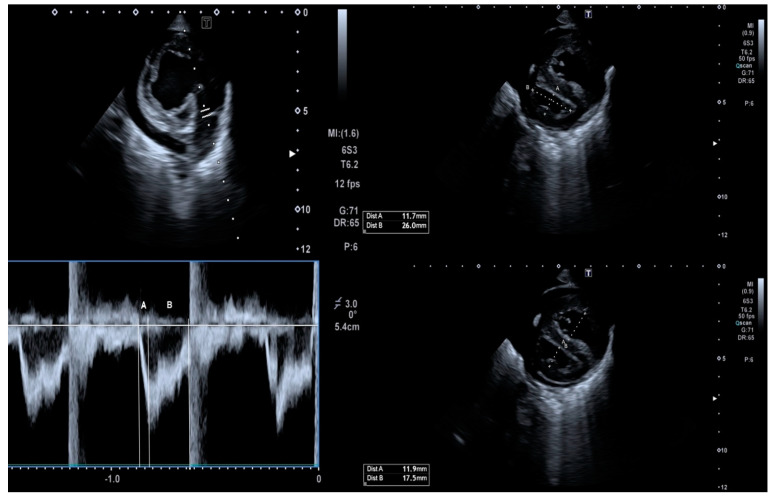
This figure shows the semiquantitative echocardiographic parameters used to assess the pulmonary artery pressures in this study. In a short axis view, we interrogated the RV outflow tract flow with pulsed-wave Doppler (left-sided image) and calculated from the resultant spectral Doppler imaging the ATET ratio (A/B). We also calculated the systolic LVEI at the level of the papillary muscles (top right-sided image) as the ratio between mediolateral and anteroposterior diameters of the LV (A/B). Finally, we calculated the RVLV ratio at the same level (B/A).

**Figure 5:**
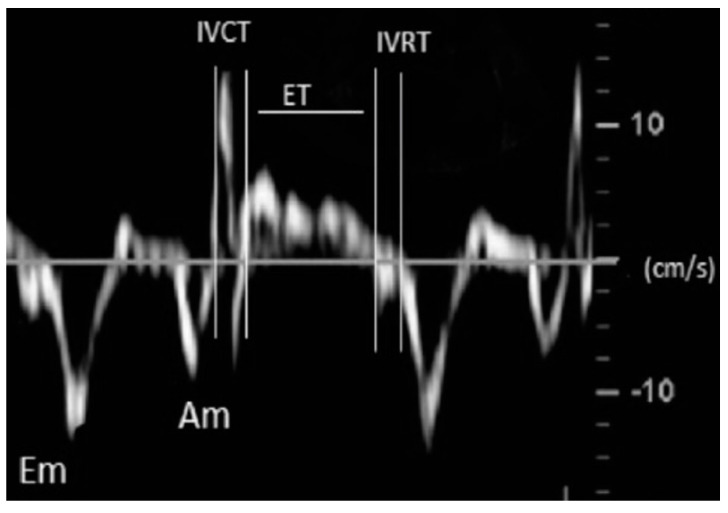
This figure shows a Doppler tissue imaging obtained at the mitral annulus for the calculation of the TEI index as a parameter for the estimation of the LV function in this study. IVCT (isovolumetric contraction time); IVRT (isovolumetric relaxation time); ET (ejection time).

One experienced pediatric cardiologist performed all echocardiographic studies following the methodology recommended in the guidelines for pediatric echocardiography [19,20,21,22,23]. Images were obtained using a Phillips IE33 ultrasound scanner (Phillips Healthcare, Madrid, Spain) with an 8 or 12-MHz sectorial transducer. Each examination was recorded, and all the studies were reviewed offline by 1 observer, who was blinded to the patient’s clinical profile. All echocardiographic measurements represented the average of 5 beats. The echocardiographic parameters used for cardiac function and pulmonary pressures evaluation included: left ventricle (LV) ejection fraction (LVEF), tricuspid annular plane systolic excursion (TAPSE), the LV and RV Tei index (LVTX and RVTX), systolic gradient of the tricuspid regurgitation jet (TRJG), RV outflow tract acceleration time-to-ejection time ratio (ATET), LV systolic eccentricity index (LVEI) and RV-to-LV ratio (RVLV) (Table 1 and Figure 4 and Figure 5).

### 2.3. Follow-Up, Data Collection and Outcome Measures

The follow-up started at the research intervention time and finished at hospital discharge. At the end of the study period, an independent observer collected the participant data through our institution’s electronic medical records and included in a relational database. The baseline information included demographic, clinical, laboratory and ultrasound data.

The existence of a significant correlation between the cardiac and respiratory parameters was the primary outcome in this investigation. The secondary endpoint was the association of different cardiopulmonary variables with PICU admission, PICU stay, the need of respiratory support higher than O_2_, the length of respiratory support and the length of stay (LOS) hospitalization.

### 2.4. Statistics

Data are presented as the median (range) or mean (standard deviation) after testing the normality with the Shapiro–Wilk test for the continuous variables and as the frequencies and percentage for the categorical variables. A Student’s *t*-test or Wilcoxon Mann–Whitney test were used to compare the mean and medians as appropriate. Proportions were compared using the chi-square test or exact methods as necessary. Spearman’s and Pearson’s coefficient tests were used to verify correlations between variables. Due to the exploratory nature of our study, significance threshold adjustments for multiple testing were not performed. Logistic regression and the receiver operator curve (ROC) analysis were used to explore the predictive accuracy of each marker for respiratory support. Odds ratio (OR), sensitivity, specificity and predictive values with 95% confidence intervals were estimated for the cut-off value at which they were considered abnormal. A significance level of 0.05 was used for all the statistical tests. Statistical analyses were performed with the use of Stata 16.0 (Stata Corp., College Station, TX, USA).

## 3. Results

### 3.1. Baseline Characteristics

We enrolled 112 infants (median age 1 (0.5–3) months; 62% males) hospitalized with acute bronchiolitis. Table 2 summarizes the baseline characteristics and clinical variables related to clinical evolution. Table 3 shows the prevalence of abnormal values of the respiratory and cardiac parameters evaluated in our population.

### 3.2. Cardiopulmonary Interactions in Bronchiolitis

Increased values of the pulmonary variables (BROSJOD score, pCO_2_ and LUS) showed moderate correlations with NT-proBNP and all the echocardiographic parameters indicative of pulmonary hypertension (TRJG, ATET, LVEI and RVLV) and global myocardial dysfunction (LVTX and RVTX) but not with the parameters of systolic myocardial dysfunction (LVEF or TAPSE) (Table 4).

### 3.3. Relationship of Cardiac and Respiratory Variables with a Severe Course of the Disease

Of the 112 (%) participants, up to 36 (32%) required respiratory support during hospitalization (Table 5). Regarding the respiratory evaluation, the respiratory support group presented with higher BROSJOD scores (*p* < 0.001), pCO_2_ (<0.001), LUS (*p* < 0.001) and lower pH levels (*p* < 0.001). With respect to the cardiac assessment, this group showed higher levels of NT-proBNP (*p* < 0.001); increased values of LVTX (*p* < 0.001), RVTX (*p* < 0.001), TRJG (*p* < 0.001), LVEI (*p* < 0.001) and RVLV (*p* < 0.001) and lower values of ATET (*p* < 0.001). All the analyzed respiratory variables and all the cardiac variables, except for LVEF and TAPSE, showed moderate-to-strong correlations with the LOS hospitalization and the time of respiratory support. None of the studied variables analyzed the presented association with the PICU stay (Table 6). All the cardiac and respiratory variables explored, except for LVEF and TAPSE, were associated with an increased risk for the need of respiratory support and also showed a moderate-to-strong predictive accuracy for the need of respiratory support in the ROC analysis, with the AUC varying from 0.74 to 0.87 (Table 7).

## 4. Discussion

In this prospective study, we employed an integrative cardiopulmonary approach to comprehensively assess the pulmonary and cardiac conditions of previously healthy infants with acute bronchiolitis at the time of hospitalization. Under normal conditions, CPI are inconsequential; however, they may become exaggerated and of great importance in certain acute pulmonary diseases. Through simultaneous clinical, laboratory and CPU assessments, we showed that a more severe respiratory state with hypoxia, respiratory acidosis and increased respiratory effort resulted in increased rates of raised pulmonary pressures and declined myocardial dysfunction, even in the absence of underlying heart disease. Of note, the presence of adverse CPI at early stages of the disease were associated with a severe course of the disease, with higher rates of respiratory support, PICU admission and prolonged hospitalizations. Therefore, the early recognition of cardiopulmonary impairment could be crucial to identifying high-risk populations for complicated acute bronchiolitis.

### 4.1. Pulmonary Status in Acute Bronchiolitis

The viral-induced inflammatory response causes peri-bronchial inflammatory infiltrate, interstitial edema and epithelial desquamation, leading to small airway obstruction, air trapping and compromise of the lung parenchyma (consolidations and atelectasis). Almost all studies in bronchiolitis describe elevated inspiratory and expiratory resistance, reduced tidal volume, increased auto-PEEP, lung hyperinflation, increased FRC and, finally, decreased respiratory compliance [24,25]. Clinically, these lung conditions manifest in the most severe cases as increased respiratory effort and impaired gas exchange with subsequent hypoxemia and acidosis. As we showed in this study, LU in infants with bronchiolitis can show diffuse and heterogeneous alveolar–interstitial involvement, which can range from mild alterations, such as pleuropulmonary line abnormalities and B-line pattern, to large pulmonary consolidations [6,7,8,12,16]. Most studies about LU in bronchiolitis used their own scores, as we did. Evaluating children with LUS, up to 25% of patients hospitalized with bronchiolitis presented moderate-to-severe pulmonary impairment. This high rate of patients with pulmonary alterations reinforces the need for LU screening early after admission. According to those previously reported, we observed a good correlation between the clinical and ultrasound findings [12,16]. Of note, LU can identify lung abnormalities not revealed by chest x-Ray [17]. Given the short time needed to get a LU report, this technique could become the routine imaging modality for the assessment of the pulmonary status in infants with bronchiolitis.

### 4.2. Cardiac Status in Acute Bronchiolitis

Although these infants are usually admitted to the hospital for the management of respiratory distress and hypoxia, the relevance of the cardiovascular involvement in these patients is increasingly documented. Cardiovascular complications are described in up to 9% of hospitalized patients [26]. Significant cardiac output and stroke volume changes have been demonstrated in infants without heart disease who were hospitalized for bronchiolitis in relation with their respiratory distress [27]. Up to 50% of patients have a positive troponin or NT-proBNP assay at the time of hospitalization. In addition, infants with bronchiolitis admitted to intensive care with hemodynamic instability and acute respiratory failure are associated with increased levels of cardiac biomarkers (cardiac troponin and NT-proBNP) compared with mild cases [28,29,30,31,32]. A recent meta-analysis showed the presence of pulmonary hypertension (pooled incidence of 20% (95% CI, 11–30%) and myocardial dysfunction (pooled incidence of 5% (95% CI, 1–9%) in 395 previously healthy infants with acute bronchiolitis [9]. Interestingly, myocardial dysfunction is frequently biventricular, as demonstrated by strain rate echocardiography (STE) in the cohort of Massolo et al. (32% of infants with bronchiolitis) [33]. Consistent with the previous evidence, approximately 20–30% of our patients developed PH and subsequent MD, and 50% presented raised NT-proBNP levels. Additionally, the presence of echocardiographic alterations correlated well with the clinical score, pH and pCO_2_. These results remark the potential utility of early point of care echocardiographic evaluation in infants hospitalized with acute bronchiolitis as a noninvasive tool to assess the severity and guide the management in a similar way to LU.

To detect myocardial dysfunction in bronchiolitis, echocardiographic study by tissue Doppler imaging (TDI) and speckle tackle echocardiography has proven to be a more sensitive method than conventional echocardiography. Tissue Doppler and STE imaging has provided more load-independent methods to assess myocardial function, with the additional advantage that high-quality two-dimensional images are no longer essential for reproducible recordings. Consistently, we observed that the TDI-derived Tei index was significantly impaired in those patients with a more compromised respiratory state. In addition, a combined systolic and diastolic measurement, such as the Tei index, may be more reflective of the overall myocardial dysfunction than those conventional methods for assessing only systolic function. Regarding the estimation of pulmonary pressures, we did not detect any differences in the pulmonary arterial pressure based on the standard TRJG findings between groups of severity. TRJG jet can be difficult to detect, and alternative semiquantitative parameters could be useful in this setting. Remarkably, the ATET, LVEI and RVLV could be measured in all infants, unlike tricuspid regurgitation, which was detectable in approximately half of them in our study. As previously reported, the inclusion of these parameters in the echocardiographic evaluation of these patients could improve the detection of PHT in this setting [9].

### 4.3. Cardiopulmonary Interactions in Acute Bronchiolitis

Our major finding was that the echocardiographic parameters assessing myocardial function and pulmonary pressures and the biomarker of myocardial strain NT-proBNP showed a moderate correlation with the respiratory clinical score that includes the respiratory rate and hypoxemia with respiratory acidosis and with our LUS. Therefore, our study documented those factors such as lung edema, consolidations and airway obstruction, leading to hypoxia, respiratory acidosis and increased respiratory effort, cause a deleterious effect in the cardiac performance in bronchiolitis. Of note, this is the first clinical study to apply a homogeneous prospective methodology investigating cardiopulmonary interactions by a combined and integrative cardiopulmonary approach in this clinically relevant setting.

The most likely underlying mechanism explaining our findings might be an increase in pulmonary vascular resistance and right heart afterload and subsequent reduced right and left ventricular functions [29,30]. Pulmonary vasoconstriction can be induced by several factors in acute bronchiolitis, including respiratory acidosis, hypoxemia, release of vasoactive inflammatory mediators, direct inflammatory endothelial damage, direct compression of pulmonary blood vessels secondary to the reduction in functional lung volume and increased alveolar pressure and a vascular remodeling phenomenon. Recent basic science studies in mouse models by Kimura et al. showed that there are structural changes in the pulmonary vasculature in bronchiolitis and that suppression of tumorigenicity 2 factor (ST2) signaling is involved in the development of RSV-associated PH, providing histopathological and pathophysiological substrates for this hypothesis [34,35].

With the increased pulmonary vascular resistance, the RV structure and function will be impaired by the increased afterload and, because of ventricular interdependence, can also cause LV myocardial dysfunction due to an abnormal pattern of relaxation and contraction secondary to geometric changes occurring in the LV [36]. In addition, the reduction in the pulmonary flow generated by RV dysfunction leads to a reduced LV preload, which is followed by a reduced stroke volume ejected by LV [37]. Furthermore, the exaggerated respiratory effort in bronchiolitis can increase the importance of these interactions. The dominant effect of a spontaneous inspiratory effort is an increased filling of the right heart and diastolic ventricular interaction with a reduced LV preload and combined increased LV afterload [3,4,38]. The increase in the work of breathing also activates the sympathetic nervous system and renin–angiotensin–aldosterone systems, contributing to increases in the ventricular afterload, altogether contributing to a decrease in LV output [3,4,38]. Additionally, the oxygen consumption is increased to values over 50% of the total oxygen consumption due to the higher activity of the diaphragm and respiratory muscles during respiratory distress [3,4,38]. Altogether, MD and reduced pulmonary and systemic CO with increased oxygen consumption could explain the sepsis-like or myocarditis-like appearance of some infants with severe bronchiolitis.

### 4.4. Predictive Accuracy of Cardiopulmonary Ultrasound for Adverse Outcomes in Acute Bronchiolitis

LU is increasingly used to assess the respiratory state and even to predict the evolution and guide the treatment of bronchiolitis, with promising results [8,12]. In the study by Bueno-Campaña et al., the ultrasound lung score combined with the WDF scale and age showed a moderate prognostic accuracy for the need of respiratory support, with an AUC of 0.84 (95% CI: 0.781–0.909), and both posterior consolidations > 1 cm and greater extension of the interstitial pattern in anterior fields presented a higher correlation with the need for respiratory support [16]. Consistently, we found that the group of patients requiring respiratory support had a worse LUS, and the 10-point LUS showed a moderate prognostic accuracy for the need of respiratory support (AUC 0.78. 95% CI 0.685–0.873) in bronchiolitis. The differences regarding accuracy could be explained by the different characteristics of the populations studied and the different LUS employed.

Echocardiography is emerging as another useful tool to assess the severity in bronchiolitis. The use of echocardiography was first applied to assess the cardiac state in children with underlying heart disease [39]. However, in recent years, there has been evidence supporting the presence of myocardial dysfunction and pulmonary hypertension also in previously healthy infants. Of note, cardiac involvement is related with the severity and prognosis of bronchiolitis (pooled relative risk = 16; 95% CI, 8.2–31.5) [9]. In our cohort, a DTI-derived Echo analysis revealed increased pulmonary pressures and reduced myocardial function in relation with a more impaired respiratory status. Beyond the predictive capacity provided by clinical and pulmonary ultrasound, most of the variables analyzed showed a moderate or good predictive value for the need of respiratory support at the cut-off value at which they were abnormal in our ROC analysis. No data about the predictive accuracy for the severity of any echocardiographic parameter was provided in previous works in this setting. Notably, the conventional echocardiographic parameters for myocardial function and pulmonary hypertension such as TAPSE, LVEF and TRJG were not useful to predict the need of PICU in this study. As LVEF and TAPSE are dependent of the ventricular geometry, preload and afterload, myocardial dysfunction could not be properly reflected by them in the acute respiratory setting, where the exaggerated respiratory efforts induced significant differences in the lung volume, leading to marked changes in the left and right ventricular loading conditions. Regarding the parameters for the detection of PHT, the ATET is a good surrogate marker for pulmonary artery pressure. ATET < 0.30 was validated against RHC to have 97% sensitivity and 95% specificity at detecting pulmonary hypertension in children [40]. In our cohort the ATET showed a good predictive accuracy for the need of respiratory support, with an AUC of 0.83 (95% CI: 0.75–0.91). Therefore, ATET could be a rapid and accurate parameter for PHT in acute bronchiolitis.

Both echocardiography and LU have been well-described as having utility for guiding the diagnosis and management of critically ill patient [10,41]. Both techniques can be performed rapidly, safely and repeatedly at point of care by the pediatrician in charge of the case. We observed that the presence of adverse CPI at early stages of the disease were associated with a severe course of the disease, with higher rates of respiratory support, PICU admission and prolonged hospitalizations. As there is no parameter with a perfect predictive capacity for bronchiolitis, our results support the use of combined CPU as a noninvasive tool to improve the early identification of patients that will require PICU admission or respiratory support. Notably, previous studies have showed that CPU has obvious advantages in investigating the etiology of acute respiratory failure in a pediatric ED setting [42]. Most unknown severe cardiac conditions in infants can mimic the presentation of bronchiolitis. Hence, infants with respiratory insufficiency from bronchiolitis may have similar clinical features to infants with congestive heart failure, presenting with irritability, fever, tachypnea, tachycardia and a mottled appearance. Therefore, we believe that a CPU approach will have broad application prospects in the initial evaluation of bronchiolitis.

### 4.5. An Integrative Approach for Severity Assessment of Acute Bronchiolitis

Although our results suggest that CPU would be a promising tool for the severity of acute bronchiolitis, we must acknowledge that some of our findings hint that less technically complex tools may perform just as well, if not better than, CPU. In this study, CPU good-quality images could be obtained from all patients who accepted to be included, but this could be due to the expertise of the investigators. Thus, one of the main limitations of the application of our results to clinical practice is the need for training to obtain adequate images in a relatively short time in infants usually irritable and with a poor acoustic window. We mentioned that clinical scores are usually limited by a high inter-observer variability. Additionally, there are many different clinical scores, making challenging the universalization of the clinical assessment of bronchiolitis in institutions. Of note, the clinical score used in this study (BROSJOD) demonstrated a correlation with the outcomes that was strongly competitive with the best ultrasonographic measures. In addition, the blood tests (NT-proBNP, pCO_2_ and pH) also performed strongly as standalone tests. The significance in distinction between the correlation coefficients of all these variables was modest (0.55 to 0.60), while the expertise required to get CPU measures varied significantly. Notwithstanding the limitation of interobserver scoring, it would likely be easier to train emergency/pediatric doctors to accurately quantify BROSJOD or to order blood test analyses as opposed to the steep learning curve and technical proficiency required for accurate CPU. Consistent with this hypothesis, we recently tested the predictive accuracy of a model including NT-proBNP, the age and the BROSJOD score with excellent results (AUC 0.945), improving the traditional assessment (age and BROSJOD score) alone [29]. Due to the exploratory nature of this work, we did not analyze predictive models here, but consistent with our previous observations, a composite model that includes components of BROSJOD with BNP and possibly pH or pCO_2_ would be strongly predictive and would require little technical up-skilling for front-line clinicians. Therefore, adding blood test analyses to the current recommendations for clinical evaluations alone seems to be an adequate approach to assess the severity in bronchiolitis pending cost efficiency studies. However, the increasing relevance and availability of point of care ultrasound in the pediatric emergency care setting joined with reluctance (based on personal experience and clinical guidelines recommendations) to perform invasive/painful blood tests in infants with bronchiolitis led us to explore a noninvasive tool such as CPU in this setting, demonstrating also a strong predictive capacity for this purpose.

All the discussions mentioned above point out that the answer to the question of what could be the best strategy for determining the severity and outcomes in bronchiolitis is complex. As all the tools analyzed present relevant limitations, maybe different combinations of them according to availability at different centers and cost efficiency issues would be the correct answer to this question. Until the best predictive model in terms of accuracy and cost efficiency is elucidated in future studies, our investigations provide evidence that there are different accurate tools available for this purpose, and each clinician/institution will choose those that best suit their local clinical practice.

## 5. Limitations

This was a single-center study with relatively few patients, so for stronger conclusions, a larger study is needed. Additionally, it may not be possible to extrapolate our findings to clinical practice in other hospitals. CPU is highly operator-dependent, and this is an inherent limitation. Echocardiographic measures and the BROSJOD score are observer-dependent but were not analyzed for intrareader or inter-reader variability in this study. All the echocardiographic parameters analyzed were already described to have high inter-reader and intrareader agreement in previous studies. There is no validated LUS in bronchiolitis, and the classification of the ultrasound findings employed in the study was developed specifically for use in this study. The NT-proBNP values are assay-dependent, and therefore, our results would not be comparable with those of centers using a different laboratory kit. Another limitation of our study was the lack of gold standard diagnostic tests for myocardial dysfunction and PHT, mainly cardiac magnetic resonance imaging and right heart catheterism. Finally, the BROSJOD score is used as a criterion for PICU admission and the initiation of respiratory support in our institution, and it could influence our results regarding its predictive accuracy for severity.

## 6. Conclusions

Our study documented that pulmonary and cardiac functions are closely connected in children hospitalized with bronchiolitis. Those patients with a worse pulmonary status presented with a more impaired cardiac status. As those patients with adverse CPI developed more severe bronchiolitis, the current findings demonstrated that CPU examination could be a useful tool with an added value to easily identify high-risk populations for complicated bronchiolitis hospitalization. Rather than compartmentalizing the two techniques, we favor the integration of echocardiography with LU in this setting, Finally, this work provided exploratory evidence about the accuracy of different tools (clinical scores, blood gases, NT-proBNP and CPU) for this purpose, and each clinician/institution will choose those that best suit their personal/local availability. Future studies are warranted to clarify the best strategy in terms of cost efficiency.

## Figures and Tables

**Table 1:** Echocardiographic parameters used to evaluate the myocardial involvement in our population.

Echo Parameter	Cardiac Parameter Evaluated	Abnormal Value	Significance
LVEF	LV systolic function	<50%	Myocardial dysfunction
TAPSE	RV systolic function	<2 SD for BSA	Myocardial dysfunction
LVTX	LV global (systolic and diastolic) function	>0.50	Myocardial dysfunction
RVTX	RV global (systolic and diastolic) function	>0.50	Myocardial dysfunction
TRJG	Pulmonary pressures	>40 mmHg	Pulmonary Hypertension
ATET	Pulmonary pressures	<0.29	Pulmonary Hypertension
LVEI	RV systolic pressure	>1.3	Pulmonary Hypertension
RVLV	RV dimension	>0.6	Dilated RV

Legend: LVEF: left ventricle ejection fraction; TAPSE: tricuspid annular plane systolic excursion; LVTX: left ventricular Tei index; RVTX: right ventricular Tei index; TRJG: tricuspid regurgitation jet gradient; ATET: acceleration time/ejection time ratio; LVEI: left ventricular eccentricity index; RV/LV: right ventricular/left ventricular ratio; BSA (Body surface area); LV (Left ventricle); RV (right ventricle).

**Table 2:** Demographic, intervention and outcome data of the study population.

Patient Number 112	Results
Age (months) *	1 (0.5–3)
Weight (kg) *	4.6 (3.7–5.8)
Gender (male) ^	62 (55)
Time from onset of symptoms to ED evaluation (days) *	1 (0–2)
RSV positive ^	86 (77)
Nebulized therapies ^	63 (58)
Oxygen therapy (nasal canulae) ^	56 (50)
Duration (days) *	2 (1–4)
HFNC ^	3 (2.5)
Duration (days) *	1 (1–2)
PICU admission ^	36 (32)
PICU stay (days) *	6 (4–9)
CPAP/BiPAP ^	28 (25)
Duration (days) *	3 (2–5)
MV ^	6 (5)
Duration (days) *	11 (10–11)
LOS hospitalization (days) *	5 (2–10)
Inotropic support	0 (0)
Death or sequel ^	0 (0)

^ Data presented in the frequency and percentage. * Data presented in the median and interquartile range. ED: emergency department. BROSJOD: Bronchiolitis Score of Sant Joan de Déu; RSV: respiratory syncytial virus; HFNC: high flow nasal cannula; PICU: pediatric intensive care unit; NIV: noninvasive ventilation; MV: mechanical ventilation; LOS: length of stay.

**Table 3:** The prevalence of abnormal or severe respiratory and the cardiac parameters evaluated in this cohort.

Patient Number (112)	N (%)
Respiratory variables	
BROSJOD score > 10 points	24 (21)
pH < 7.30	30 (26)
pCO_2_ > 55 cmH20	51 (45)
LUS > 10 points	25 (22)
Cardiac variables	
NT-proBNP > 1121 pg/mL	57 (51)
Myocardial function	
LVEF < 50%	0 (0)
TAPSE < 2 Z-score for BSA	4 (3.5)
LVTX > 0.5	26 (23)
RVTX > 0.5	25 (22)
Pulmonary pressures	
TRJG < 40 mmHg (*n* = 50)	11 (22)
ATET < 0.29	31 (27)
LVEI > 1.3	31 (27)
RVLV > 0.6	39 (35)

Legend: BROSJOD: Bronchiolitis Score of Sant Joan de Déu; LUS: lung ultrasound score; NT-proBNP: N-Terminal Pro-Brain Natriuretic Peptide; LVEF: left ventricle ejection fraction; TAPSE: tricuspid annular plane systolic excursion; LVTX: left ventricular Tei index; RVTX: right ventricular Tei index; TRJG: tricuspid regurgitation jet gradient; ATET: acceleration time/ejection time ratio; LVEI: left ventricular eccentricity index; RV/LV: right ventricular/left ventricular ratio; BSA: body surface area.

**Table 4:** Correlation of the cardiac variables with the respiratory variables studied in this investigation.

Cardiac Variables	BROSJOD Score		pCO_2_		LUS Score	
	CC	*p*-Value	CC	*p*-Value	CC	*p*-Value
NT-proBNP (pg/mL) *	0.37	0.001	0.48	0.000	0.46	0.000
LVEF (%) *	0.01	Ns.	−0.05	Ns.	−0.05	Ns.
LVTX **	0.25	0.006	0.39	0.000	0.33	0.000
TAPSE *	−0.10	Ns.	−0.18	Ns.	−0.19	Ns.
RVTX *	0.35	0.000	0.44	0.000	0.37	0.000
TRJG (mmHg) *, *n* = 50	0.36	0.010	0.56	0.000	0.34	0.014
ATET *	−0.37	0.001	−0.43	0.000	−0.34	0.000
LVEI *	0.38	0.000	0.48	0.000	0.45	0.000
RVLV *	0.26	0.005	0.30	0.002	0.32	0.000

*n*: sample size. CC: Spearman’s (*) or Pearson (**) correlation coefficient. BROSJOD: Bronchiolitis Score of Sant Joan de Déu; LUS: lung ultrasound score.; NT-proBNP: N-Terminal Pro-Brain Natriuretic Peptide; LVEF: left ventricle ejection fraction; LVTX: left ventricular Tei index; TAPSE: tricuspid annular plane systolic excursion; RVTX: right ventricular Tei index; TRJG: tricuspid regurgitation jet gradient; ATET: acceleration time/ejection time ratio; LVEI: left ventricular eccentricity index; RV/LV: right ventricular/left ventricular ratio; Ns.: *p* > 0.05.

**Table 5:** Respiratory and cardiological evaluation according to the need for respiratory support during hospitalization.

Variable	Respiratory Support (*n* = 36; 32%)	No Respiratory Support (*n* = 76; 68%)	*p*-Value
**Demographic and outcome data**			
Age (months) *	1 (0.5–2.5)	1 (0.5–3)	Ns.
Weight (kg) *	4.3 (3.5–5.8)	4.8 (4–6.2)	Ns.
Gender (male) ^	19 (53)	43 (56)	Ns.
Time of symptoms previous admission (days) *	1 (0–2)	1 (0–2)	Ns.
RSV positive ^	32 (89)	54 (71)	Ns.
**Pulmonary assessment**			
BROSJOD score (points) *	11 (9–13)	6 (4–8)	0.000
pH ^**	7.28 (0.07)	7.34 (0.05)	0.000
pCO_2_ (cmH_2_O) *	62 (47–80)	47 (42–54)	0.000
LUS (points) *	10 (5–13)	4 (1–6)	0.000
**Cardiac assessment**			
NT-proBNP (pg/mL) *	3949 (2249–6042)	722 (309–1461)	0.000
LVEF (%) **	39.7 (2.4)	39.6 (2.6)	Ns.
LVTX **	0.60 (0.17)	0.41 (0.10)	0.000
TAPSE **	14 (2.5)	15 (2.2)	Ns.
RVTX **	0.59 (0.12)	0.42 (0.10)	0.000
TRJG (mmHg) **, *n* 50	38 (14)	25 (9.5)	0.000
ATET **	0.28 (0.05)	0.37 (0.06)	0.000
LVEI **	1.36 (0.21)	1.1 (0.13)	0.000
RVLV **	0.62 (0.12)	0.49 (0.10)	0.000

^ Data presented in the frequency and percentage. * Data presented in the median and interquartile range. ** Data presented in the mean (standard deviation). Ns.: *p* > 0.05. RSV: respiratory syncytial virus. BROSJOD: Bronchiolitis Score of Sant Joan de Déu; LUS: lung ultrasound score; NT-proBNP: N-Terminal Pro-Brain Natriuretic Peptide; LVEF: left ventricle ejection fraction; LVTX: left ventricular Tei index; TAPSE: tricuspid annular plane systolic excursion; RVTX: right ventricular Tei index; TRJG: tricuspid regurgitation jet gradient; ATET: acceleration time/ejection time ratio; LVEI: left ventricular eccentricity index; RV/LV: right ventricular/left ventricular ratio.

**Table 6:** Correlation of the cardiac and respiratory variables with the outcome data.

Cardiac and Respiratory Variables	LOS Hospitalization		PICU Stay		Duration of RS	
	CC	*p*-Value	CC	*p*-Value	CC	*p*-Value
BROSJOD score *	0.55	<0.001	−0.08	Ns.	0.47	<0.001
pH *	−0.34	<0.001	−0.21	Ns.	−0.23	0.021
pCO_2_ *	0.42	<0.001	0.03	Ns.	0.29	0.004
LUS *	0.58	<0.001	0.06	Ns.	0.02	Ns.
NT-proBNP *	0.60	<0.001	0.14	Ns.	0.50	<0.001
LVEF *	0.01	Ns.	−0.05	Ns.	−0.05	Ns.
LVTX *	0.49	<0.001	0.17	Ns.	0.40	<0.001
TAPSE *	−0.10	Ns.	−0.18	Ns.	−0.19	Ns.
RVTX *	0.58	<0.001	0.23	Ns.	0.42	<0.001
TRJG, *n* = 50 *	0.48	<0.001	−0.13	Ns.	0.31	0.024
ATET *	−0.52	<0.001	−0.18	Ns.	−0.45	<0.001
LVEI *	0.58	<0.001	0.20	Ns.	0.44	<0.001
RVLV *	0.54	<0.001	0.21	Ns.	0.38	<0.001

*n*: sample size. CC: Spearman’s (*) or ** Pearson (**) correlation coefficient. LOS: length of stay; PICU: pediatric intensive critical care unit; RS: respiratory support; BROSJOD: Bronchiolitis Score of Sant Joan de Déu; LUS: lung ultrasound score; NT-proBNP: N-Terminal Pro-Brain Natriuretic Peptide; LVEF: left ventricle ejection fraction; LVTX: left ventricular Tei index; TAPSE: tricuspid annular plane systolic excursion; RVTX: right ventricular Tei index; ATET: acceleration time/ejection time ratio; LVEI: left ventricular eccentricity index; RV/LV: right ventricular/left ventricular ratio. Ns.: *p* > 0.05.

**Table 7:** Predictive value of different cardiopulmonary parameters for the need of advanced respiratory support.

Predictive Variables	OR (95% CI)	AUC (95% CI)	S	Sp	PPV	NPV
BROSJOD > 10 points	28 (12–125)	0.87 (0.78–0.93)	0.91	0.61	0.91	0.76
pH < 7.30	3.5 (1.5–8.5)	0.72 (0.61–0.83)	0.44	0.81	0.53	0.75
pCO_2_ > 55 cmH_2_O	4.3 (2–10)	0.74 (0.62–0.86)	0.69	0.65	0.49	0.82
LUS > 10 points	6 (2.3–15)	0.77 (0.67–0.85)	0.44	0.88	0.64	0.77
NT-proBNP > 1121 pg/mL	9 (3.3–24)	0.83 (0.73–0.91)	0.85	0.71	0.55	0.90
LVTX > 0.5	8.5 (3–22)	0.80 (0.69–0.89)	0.50	0.89	0.70	0.79
RVTX > 0.5	18 (5.8–54)	0.84 (0.76–0.92)	0.55	0.93	0.80	0.81
LVEI > 1.3	15 (5.5–40)	0.84 (0.75–0.92)	0.64	0.89	0.74	0.84
ATET < 0.29	7.4 (3–18)	0.83 (0.75–0.91)	0.55	0.85	0.65	0.80
RVLV > 0.6	8.4 (3.3–21)	0.77 (0.68–0.86)	0.61	0.84	0.64	0.82

AUC: Area under the receiver operator curve; S: sensitivity; Sp: specificity; PPV: positive predictive value; NPV: negative predictive value; BROSJOD: Bronchiolitis Score of Sant Joan de Déu; LUS: lung ultrasound score; NT-proBNP: N-Terminal Pro-Brain Natriuretic Peptide; LVTX: left ventricular Tei index; RVTX: right ventricular Tei index; LVEI: left ventricular eccentricity index; ATET: acceleration time/ejection time ratio; RV/LV: right ventricular/left ventricular ratio.

## Data Availability

The data presented in this study are available within this article.
